# The effect of carotid sinus neurectomy for carotid restenosis: a study protocol for a double-blinded and randomized controlled trial

**DOI:** 10.1186/s13063-023-07871-3

**Published:** 2024-01-09

**Authors:** Zhi Zhang, Xiang Ji, Yihao Tao, Ning Huang, Rong Wen, Jun Tang, Yuan Cheng, Zongyi Xie, Guodong Liu, Guanjian Zhao

**Affiliations:** 1 Department of Neurosurgery, The Second Affiliated Hospital of Chongqing Medical University, Chongqing, 400010 China; 2 Department of gynaecology and obstetrics, The second Affiliated Hospial of Chongqing Medical University, Chong Qing, China

**Keywords:** Carotid endarterectomy, Carotid sinus nerve, Carotid artery restenosis, Inflammation, Randomized controlled trial

## Abstract

**Background:**

Patients undergoing carotid endarterectomy (CEA) have a high restenosis rate, which increases the risk of stroke, and there is still a lack of effective treatment for restenosis. The cause of stenosis is related to local inflammatory reactions. Some basic studies have shown that the inflammatory response causing arterial stenosis is closely related to the nerve axons distributed in its outer membrane, and that removal of the nerve is effective in reducing the inflammatory response to prevent arterial stenosis. Therefore, we propose to design a randomized controlled trial to study whether disconnecting the carotid sinus nerve during a CEA operation can reduce carotid arterial restenosis.

**Method/design:**

This study is a randomized, double-blind, single-center study. We will recruit 276 patients, who will be randomly divided into the experimental group and the control group. Based on the standard CEA operation, the operator will search for the carotid sinus nerve on the surface of the internal carotid artery and will entirely transect it in the experimental group. Both groups will be guided with the same postoperative treatment and will be followed up every 3 months for 3 years after the operation. The main indices observed will be the carotid restenosis rate, incidence and nature of carotid plaque, and carotid blood flow velocity. Other indices will be arrhythmia, blood pressure variability, and biomarkers of atherosclerosis, such as blood lipids, hypersensitive C-reactive protein (hs-CRP), homocysteine, and total bilirubin.

**Discussion:**

It is expected that carotid sinus nerve transection will significantly reduce the occurrence of restenosis after CEA, decrease the incidence of ischemic stroke, and realize the effective primary prevention of stroke.

**Trial registration:**

ChiCTR2300073652. Registered on July 18, 2023.

**Supplementary Information:**

The online version contains supplementary material available at 10.1186/s13063-023-07871-3.

## Introduction

Carotid artery stenosis (CAS) is characterized by a high prevalence and high disability rates. A meta-analysis in America showed that the overall prevalence rate of carotid artery stenosis ≥ 50% is 4.2% [1]. In China, the proportion of adults with CAS ≥ 50% by ultrasound examination is as high as 7%, and its younger trend is gradually becoming apparent, with the 40- to 64-year-old workforce accounting for 50% [2]. CAS, as an essential factor causing stroke, has attracted much attention. Studies have shown that CAS causes approximately 2530% of strokes. According to World Health Organization statistics, stroke is second only to heart disease, the leading cause of death worldwide [3].

Currently, the surgical methods for severe CAS include CEA and carotid artery stent implantation (CASI). However, patients who underwent CEA and CASI could have restenosis after the operation. It has been reported that the probability of carotid restenosis is still 5–22% even if the best drug treatment is adopted after the process [4–6]. Restenosis is usually defined as stenosis with a stenosis degree of more than 50% at a time period longer than 1 month after the operation. Compared with the first stenosis, treating patients with carotid restenosis is more challenging, and it may be dangerous to intervene in these high-risk patients again [7]. Because of the existence of scar tissue, it is usually challenging to perform CEA again because it increases the cranial nerve injury incidence. Some patients are also treated with CAS for restenosis after CEA, but the risk of recurrence after CASI treatment is more significant than that after CEA again. There is currently no clear suggestion for the treatment of restenosis. Some studies have tried to use angioplasty, carotid artery bypass, and other treatment measures [8, 9], but there is still a lack of prospective research.

It is believed that the early causes of restenosis after CEA are abnormal proliferation of the vascular endothelium and vascular remodeling. In CEA surgery, an endothelial injury occurs. Then, neointimal hyperplasia is caused by several cell-mediated chemokines, such as inflammatory cells and smooth muscle cells, which promote the pathological process characterized by narrowing blood vessels [10]. The cause of restenosis in the middle and late stages is similar to that of primary CAS, and more than 90% of restenosis is caused by carotid atherosclerosis [11].

Numerous studies have shown that the immune system participates in the formation and progression of atherosclerotic plaques by inducing leukocyte infiltration in the adventitia of arteries [12–14]. The progression of atherosclerosis is parallel to the aggregation of immune cells in the adventitia of adjacent plaques. The absence of such a collection of immune cells in the adventitia of nonplaque parts of arteries also indicates that the immune response plays a vital role in the process of atherosclerosis [15]. Andreas et al.’s research further showed that the aggregation of inflammatory cells in the plaque site is mediated by nerve axons distributed on the adventitia of this site. They found that the activity of the splenic sympathetic nerve and the abdominal vagus nerve is parallel to the progression of abdominal aorta atherosclerosis, and celiac ganglion resection can inhibit the immune response in the corresponding area of the abdominal aorta, thus inhibiting disease progression and promoting plaque stability [16].

The most easily formed part of carotid plaque is the end of CAS, which is the bifurcation of the internal carotid artery and external carotid artery. Compared with other parts, there are more nerves distributed in the adventitia of this part, and these nerves are composed of many terminal nerves from carotid sinus nerves. The carotid sinus nerve originates from the glossopharyngeal nerve, runs in front of the carotid artery approximately 1–3 cm after it exits the jugular foramen, and terminates in the carotid sinus, carotid body, and the interscalene, with a total length of approximately 4–8.8 cm and a diameter of approximately 0.5–1.5 mm [17]. The carotid sinus and carotid body are pressure receptors and chemoreceptors, respectively. Stimulating the carotid sinus has a sympathetic inhibitory effect on angiogenesis (blood pressure lowering) and the heart (bradycardia). After activation, the latter produces hyperventilation and promotes stimulation of the sympathetic nerve output of blood vessels (blood pressure increase) and parasympathetic nerve activity of the heart (bradycardia). In the treatment of carotid sinus syndrome, bilateral carotid sinus adventitia peeling has been used to inactivate carotid sinus nerves. This method has been shown to be safe and effective. Compared with the control group, there was no significant difference in blood pressure, blood pressure variability, or carotid diameter [18].

We assume that during CEA, the carotid sinus nerve on this side should be searched without enlarging the incision. It should be entirely transected to inactivate the nerves distributed in the carotid adventitia to block the inflammatory reaction mediated by the nerves at this part, prevent intimal hyperplasia and plaque formation after CEA, and control the occurrence of restenosis.

## Methods/design

### Study design

**Fig. 1:**
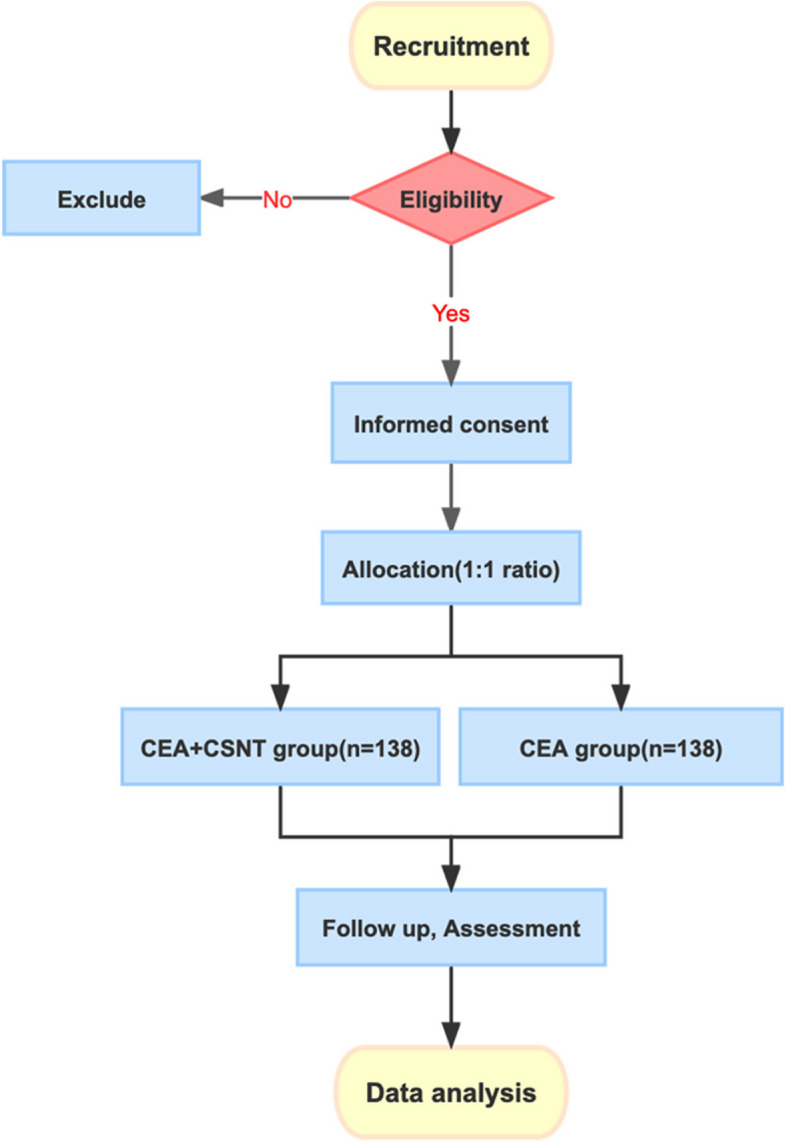
Trial flow chart

This prospective, randomized, double-blind, single-center, superiority clinical trial will be conducted at the Second Affiliated Hospital of Chongqing Medical University in China. The study was registered with the Chinese Clinical Trial Registry on 18 July 2023 (ChiCTR2300073652). The plan is to recruit 276 eligible subjects and assign them to either the control group (standard CEA therapy) or the experimental group (CEA+ carotid sinus nerve transection, CSNT) at a 1:1 ratio. Participants will be hospitalized for 2 weeks and will have a 3-year follow-up period. The trial flow chart is shown in Fig. 1.

### Recruitment and eligibility criteria

Enrollment will be completed within 3 years (from the beginning of recruitment to the last patient). Enrollment is projected to begin on July 18, 2023. Recruitment advertisements for the study will be placed on the WeChat public website and hospital websites. The research staff (Y. H. T., X. J., J. T., N. H., and R. W.) will screen inpatients and outpatients according to the inclusion and exclusion criteria.

The inclusion criteria are as follows: (1) aged 55–80 years, female or male; (2) symptomatic carotid artery stenosis and noninvasive examination showed that the stenosis of the carotid artery was ≥ 70%, or angiography showed that the stenosis exceeded 50%; (3) internal carotid artery was not completely occluded; (4) National Institutes of Health Stroke Scale (NIHSS) scores ≤ 4; and (5) the patient and his or her family members were informed and provided signed and informed consent before surgical treatment. The exclusion criteria are as follows: (1) CEA or CAS surgery for unilateral or bilateral; (2) clinical status complicated by severe organ system diseases such as renal failure, heart failure, and liver cirrhosis; (3) serious psychological disease; and (4) severe arrhythmia or carotid sinus syndrome.

The withdrawal criteria are as follows: (1) voluntary withdrawal during the intervention, (2) alcohol and/or drug abuse, and (3) any other reasons considered inappropriate by the investigators.

### Outcomes

1. Carotid artery restenosis rate

Restenosis is defined as a degree of carotid artery stenosis ≥ 50% by ultrasound examination after CEA, and its proportion in CEA patients is the restenosis rate [1].

2) Incidence and properties of carotid plaque

The proportion, size, thickness, and stability of the carotid arterial plaque

3) Carotid artery blood flow velocity

### Other measures

These are arrhythmia; blood pressure variability; biomarkers for atherosclerosis, such as hs-CRP, homocysteine, and total bilirubin; and blood lipids, such as triglycerides, total cholesterol, high-density lipoprotein, low-density lipoprotein, and nonesterified fatty acids [19].

### Interventions

The patients will be allocated to either the experimental or control group based on a blocked randomization sequence generated by SPSS 23.0 software for Windows.

### Surgical treatment

All patients will be treated with a CEA standard operation. After general anesthesia, the patient will be placed in the supine position, with the shoulder pad, head and neck facing the contralateral hyperextension position, and the basal blood pressure level will be maintained. During the operation, the changes in intracranial blood flow will be monitored by transcranial Doppler. The carotid artery and its branches will be dissected along the incision at the front edge of the sternocleidomastoid muscle. If the patient’s carotid artery bifurcation is high or the lesion area is extended, the digastric power can be cut off to block the distal end of the internal carotid artery. After dissecting the bifurcation of the common carotid artery, internal carotid artery, external carotid artery, and superior thyroid artery clearly, a blocking band will be sleeved, and appropriate blocking forceps will be selected for later use. After the preparation is finished, whole body heparin will be given at 1 mg/kg, and 1% lidocaine will be used for carotid sinus infiltration anesthesia. To avoid excessive blood pressure fluctuation, a longitudinal incision will be made to cut the blood vessel, peel off the plaque, and remove it as altogether as possible. Heparin saline will be used to wash the peeling surface, and the intimal flap will be floated to fix it. The blood vessel will be sutured, the blocking forceps will be loosened, and the blood pressure will be lowered by 10–20% of the basal blood pressure to reduce the risk of postoperative overperfusion. The subcutaneous tissue and skin will be sutured in turn. After suturing, the coagulation function will be checked urgently, and whether to use protamine to neutralize heparin will be decided according to the results.

In the experimental group, after suturing the blood vessels, the carotid sinus nerve will be searched for along the front of the internal carotid artery, separated and exposed approximately 1 cm in length to be entirely transected, and then the subcutaneous and skin will be sutured layer by layer.

### Postoperative treatment

Statins (double dose) will be used to strengthen lipid regulation and stabilize the plaque. Aspirin (100-mg QD) combined with clopidogrel (75-mg QD) will be utilized for antiplatelet therapy for 1 month, and then statins (conventional dose) and aspirin (100-mg QD) will be used for antiplatelet therapy for a long time.

### Baseline data collection

We will request consent for review of participants’ medical records and for the collection of blood samples to assess blood lipids, hs-CRP, homocysteine, liver function, and kidney function within 1 week before the operation [20]. The blood samples after inspection will be stored in special liquid nitrogen tanks for future use in auxiliary research. On the second day after the procedure, high-resolution-enhanced magnetic resonance imaging (MRI) will be reexamined to understand the recovery of the carotid canal cavity and plaque clearance for subsequent comparison. Carotid artery color Doppler ultrasound will be performed 1 week after the operation to understand plaque.

### Follow-up

Follow-up in the outpatient department will be performed every 3 months. At the same time, blood lipids, hs-CRP, homocysteine, liver function, and kidney function will be reexamined after obtaining informed consent. Carotid artery color Doppler ultrasound and carotid artery high-resolution enhanced MRI will be reviewed at 3 months, 6 months, 1 year, 2 years, and 2 years after the operation.

### Sample size

The determination of sample size was calculated by SPSS 23.0 software. Based on a previous study and clinical assumptions, carotid restenosis was 5–22% [4–6]. Our previous research showed that the restenosis rate of the carotid artery after CEA in our hospital was approximately 15%. The statistical parameters were set as follows: one-sided log-rank testing with 80% power, the probability of obtaining a false-positive with a statistical test at 0.05, and a 3-year follow-up time. Considering a potential dropout rate of 5% across both groups, it is estimated that 276 patients will need to be enrolled (138 participants per group).

### Randomization and blinding

Eligible subjects will be randomly divided into two parallel pairs of groups in a 1:1 ratio according to a software-generated random sequence (produced using SPSS software). Before the study group assignment, the allocation sequence will be concealed using sealed, opaque, and stapled envelopes that the participants or recruiters will not open. The sequence generation and allocation concealment will be implemented by a researcher who will not be involved in the recruitment process. A specific investigator will be responsible for processing the study group assignments.

The participants and outcome assessors will remain blind to the group allocation until the end of the trial. The study data statistician will be unaware of the assignments and will not participate in the follow-up visit. Regular unblinding will be performed by the principal investigator and statistical experts for the first time to conduct statistical analysis according to the statistical plan. The researchers will complete a second blinding process to determine which of the two groups is the experimental group. To prevent unnecessary unblinding or harm, emergency unblinding of allocation will be performed if patients meet the following criteria: (1) severe violation of the treatment plan, (2) severe adverse events during the intervention, (3) the patients or their family members requesting to stop the trial, and (4) visitors who are lost to follow-up.

### Data collection and management

A case report form (CRF) will be completed by each participant before the intervention. The collection of baseline data, along with primary and secondary outcomes, will be recorded entirely in the CRF. Each participant (corresponding to a CRF) will be assigned a specific number by which the intervention and follow-up will be performed. Using ResMan, an Internet-based electronic data capture (EDC) tool, all information will be kept independently as double copies in a computer. The data files will be locked after a blind audit and when they are confirmed reliable. The CRF shall be owned by the principal investigator and shall not be provided to any third party in any form without the written approval of the principal investigator, who will oversee all of the final trial data. A follow-up registration form will be given to all patients to encourage retention. A researcher will collect the reason for the loss, survival condition, and recent outcome as an assessment for participants who stop the intervention. The original paper files will be kept in the filing cabinet of the sponsoring organization, and clinical data will be held for 5 years.

### Quality control

#### Composition of the coordinating center and trial steering committee

The trial will be performed by the neurosurgery of the Second Affiliated Hospital of Chongqing Medical University. There is no other coordinating center. The trial steering committee consists of the ethics committee of the Second Affiliated Hospital of Chongqing Medical University (ID: LKSD-2023-88) and the clinical research team. The research team consists of three principal investigators who oversee the study and are responsible for medical responsibilities, a study coordinator who plans patient visits, and an investigator who is responsible for data management and trial management. The research team meets monthly to assess the progress, identify potential test subjects, and work out logistical issues. A statistician was involved in the design of the trial and will be consulted on statistical issues throughout the study. Guodong Liu, Yuan Cheng, and Zongyi Xie will identify the enrolled patients in the outpatient department. A researcher will collect general patient information. To improve the reliability of the test and reduce the differences between operators, the following measures will be taken: all the operations will be led by Guodong Liu, and all the operations will be photographed and recorded by video. The test data will be evaluated and counted by one person.

A data monitoring committee (DMC), consisting of neurosurgery experts and statisticians, will be established, independent of the sponsor and trial investigator. The DMC will assess the progress of the clinical trials, safety data, and essential efficacy data yearly. The DMC will assess the progress of the clinical trials, safety data, and essential efficacy data yearly. The REC meeting will also assess the ethics of the trial every year.

In the study, an inspector will verify the accuracy and completeness of the CRFs and compare them with the source documents yearly. During inspections, it must be ensured that the subject’s dose changes, treatment changes, adverse events, combined medications, missed visits, and examination omissions are noted in the CRFs. Additionally, they must confirm that the withdrawals and missed visits of the selected subjects have been recorded and explained in the CRFs. The inspector will also notify the researcher of errors, omissions, or unclear hand-writing in CRFs and ensure that the researcher or authorized person carries out a correction, addition, or deletion. The modification must be documented in writing, if necessary. The inspector will promptly inform the principal after each supervision and inspection. The experimental results will be published in relevant publications for public access.

### Statistical analysis

We will use SPSS 23.0 statistical software for Windows to perform statistical analysis. To ensure the reliability of our conclusions, we plan to use intention-to-treat (ITT) analysis as the primary form of research.

### Baseline description

Means ± standard deviations (SD) will be used to describe normally distributed quantitative data; medians and interquartile ranges (IQRs) will be used to describe nonnormal distribution data. Blood lipid, hs-CRP, homocysteine, liver function, and kidney function data will be determined if it is normally distributed or not to decide whether to use SDs or IQRs. The constituent ratio or relative ratio will be used to describe the count data (recovery of the carotid canal cavity and plaque clearance).

### Comparison of baseline data

*T*-tests will be performed for quantitative data that are usually distributed, and a nonparametric test will be used for quantitative data that are not normally distributed, including blood lipids, hs-CRP, homocysteine, liver function, and kidney function. The chi-squared (*χ*^2^) test will be used for counting data, including recovery of the carotid canal cavity and plaque clearance. A two-sided *P*-value < 0.05 will be considered statistically significant.

### Interim analysis

During the randomized controlled trial (RCT), one safety check will be performed when 50% of the needed patients are enrolled and the 3-year follow-up has been completed for these patients. The data will be shared by the program coordinator with the DMC, who will perform the interim analysis. The DMC is not involved in the direct implementation of the treatment. If a statistically significant difference is observed and the carotid restenosis rate in the experimental group is higher than that in the control group, the trial will be ended; otherwise, the trial will be kept secret from the researcher and continue as planned in both groups.

### Comparison of therapeutic efficacy

The rate of carotid artery restenosis, plaque incidence, and stability of carotid arterial plaque will be analyzed by the chi-square (*χ*^2^) test between the two arms.

The size and thickness of carotid arterial plaque, carotid artery blood flow velocity, blood lipids, hs-CRP, homocysteine, and total bilirubin will be analyzed by *t*-test analysis.

### Safety evaluation

The incidence of adverse events (arrhythmia, blood pressure variability) will be expressed as the number of patients (percentage) and analyzed by the chi-square (*χ*^2^) test.

### Reports of adverse events

Adverse events (AEs) will be assessed primarily by electrocardiogram and blood pressure monitoring. The occurrence, severity level, management strategies, causality related to the experimental surgery, and termination of all adverse events (AEs) will be recorded and preserved during the clinical trials and follow-up. All severe adverse events (serve arrhythmia) will be reported promptly to the principal investigator and the research ethics committee (REC). The researchers and clinical trial institutions will ensure that the subjects are treated appropriately and informed truthfully of the relevant information. According to China’s Management Specification for Classification and Sort of Surgical and Invasive Procedures in Medical Institutions, researchers are responsible for the cost of dealing with adverse reactions and compensation for patients who suffer adverse reactions. Participants are not provided with specific posttrial care (Table 1).

**Table 1:** The flow diagram of enrollment, allocation, interventions, and follow-up for participants

	Enrollment	Allocation	Intervention		Follow-up				
			CEA	neurectomy	3 M	6 M	1 Y	2 Y	3 Y
Timepoint	1 week	0	1 week		3 years				
Enrollment									
Eligible screen	×								
Informed consent	×								
Demographic & medical data	×								
Random allocation		×							
Interventions									
Experimental group			×	×					
Control group			×						
Assessments									
Color Doppler ultrasound	×				×	×	×	×	×
HRMR	×				×	×	√	√	×
ECG	×				×	×	×	×	×
Biomarkers for atherosclerosis	×				×	×	×	×	×
Adverse events					×	×	×	×	×

*CEA* carotid endarterectomy, *HRMR* high-resolution enhanced nuclear magnetic resonance, *ECG* electrocardiogram, *M* month, *Y* years

## Discussion

There is considerable controversy about the treatment of restenosis after CEA, and there is a lack of relevant guidelines. Doctors must rely on the degree of stenosis, clinical symptoms, and vascular compensation to make clinical decisions [11]. Some methods are used to perform CEA surgery or CASI treatment again. Nevertheless, according to different research reports, it may be dangerous to perform CEA or CASI on restenosis patients [7]. Scar tissue makes it more challenging to redo CEA, and CASI cannot achieve an excellent therapeutic effect. A study showed that the probability of intrastent restenosis in patients treated with CASI may reach 24% within 4 years [21].

Before this, carotid sinus denervation was used to treat carotid sinus syndrome, hypertension, heart failure, and other diseases, and its safety has been confirmed. In the treatment of carotid sinus syndrome, researchers found that the carotid sinus can be denervated by peeling off the adventitia 3 cm from the proximal end of the internal carotid artery. By injecting lidocaine into the carotid sinus for anesthesia, the blood pressure and heart rhythm of patients can be kept stable during the operation, with no significant fluctuation in systolic blood pressure, diastolic blood pressure, or heart rhythm observed after the process [19]. Others have shown that removing the carotid sinus nerve for the treatment of essential hypertension will not reduce the blood pressure of rats, while removing the bilateral carotid sinus nerve can reduce the blood pressure of hypertensive rats by 20 mmHg, which also shows that unilateral carotid sinus nerve removal will not affect blood pressure in this study [22].

A consensus has been reached regarding inflammation-mediated intimal hyperplasia, vascular reconstruction, and plaque formation. However, after CEA, the best medical therapy, including inhibition of inflammation, still causes restenosis in many patients [23]. The latest research shows that although there is no nerve distribution in the intima of the blood vessel, the inflammatory reaction in this part is closely related to the nerve axons distributed on the adventitia. By removing this axon, the local inflammatory response can be eliminated, thus effectively inhibiting plaque formation and blocking the arterial stenosis caused by it [16]. Carotid stenosis mainly occurs near the bifurcation, which is just a nerve-rich area, and a small branch distribution of a single carotid sinus nerve. Simply cutting the carotid sinus nerve can achieve the effect of removing the nerve distribution.

We will use the carotid restenosis rate as the main index of the study, and at the same time, we will also select the incidence and properties of carotid plaque; carotid artery blood flow velocity arrhythmia; blood pressure variability; biomarkers for atherosclerosis, such as hs-CRP, homocysteine, and total bilirubin; and blood lipids, such as triglycerides, total cholesterol, high-density lipoprotein, low-density lipoprotein, nonesterized fatty acid, and other research indicators, to comprehensively evaluate the factors related to carotid artery blood supply and plaque formation. Follow-up began in March after the operation and will last for up to 3 years, which is consistent with most of the studies on carotid restenosis carried out at present [4–9]. Therefore, this study intends to observe the carotid restenosis rate, plaque formation, and other indicators after disconnecting the carotid sinus nerve in standard CEA surgery to confirm that this operation can effectively reduce the incidence of carotid restenosis after CEA.

### Trial status

The trial started on July 18, 2023, and recruited 13 patients in our hospital. The trial was started according to the protocol in its second version dated March 23, 2023. There were minor protocol modifications after starting the trial (versions 3.0, date July 30, 2023); the only change was that the total sample size was revised to 276 patients, and this change had no impact on the study conduct. According to the planned timeframe, recruitment will be ongoing until March 31, 2024, and patient follow-up is expected to be completed by March 31, 2027. Upon completion, the results of the trial will be shared in an open-access journal. The protocol modifications will be submitted to the REC and the Chinese Clinical Trial Registration authority for review and record by website uploading.

### Supplementary Information

**Additional file 1.** SPIRIT 2013 Checklist. Recommended items to address in a clinical trial protocol and related documents.

## Data Availability

Please contact the corresponding author: 304867@hospital.cqmu.edu.cn or visit http://www.medresman.org.cn/pub/cn/proj/search.aspx.

## Abbreviations

AEs: Adverse events
CEA: Carotid endarterectomy
CAS: Carotid artery stenosis
CASI: Carotid artery stent implantation
CSNT: Carotid sinus nerve transection
CRF: Case report form
DMC: Data monitoring committee
EDC: Electronic data capture
ECG: Electrocardiogram
hs-CRP: Hypersensitive C-reactive protein
HRMR: High-resolution enhanced nuclear magnetic resonance
ITT: Intention to treat
IQRs: Interquartile ranges
MRI: Magnetic resonance imaging
M: Month
NIHSS: National Institutes of Health Stroke Scale
RCT: Randomized controlled trial
REC: Research ethics committee
SD: Standard deviation
Y: years
